# Combined therapy of Ulmo honey (Eucryphia cordifolia) and ascorbic acid to treat venous ulcers^1^

**DOI:** 10.1590/0104-1169.0020.2550

**Published:** 2015

**Authors:** Mariano del Sol Calderon, Carolina Schencke Figueroa, Jessica Salvo Arias, Alejandra Hidalgo Sandoval, Felipe Ocharan Torre

**Affiliations:** 2 PhD, Full Professor, Facultad de Medicina, Universidad de La Frontera, Temuco, Chile; 3 Doctoral student, Universidad de La Frontera, Temuco, Chile. Scholarship holder from Universidad de La Frontera, Temuco, Chile; 4 MSc, Assistant Professor, Facultad de Medicina, Universidad Mayor, Temuco, Chile; 5 RN, Hospital Makewe-Pelale, Temuco, Chile; 6 Midwife, Hospital Makewe-Pelale, Temuco, Chile

**Keywords:** Varicose Ulcer, Honey, Ascorbic Acid, Combined Modality Therapy

## Abstract

**OBJECTIVE:**

to assess the clinical effect of topical treatment using Ulmo honey associated with oral ascorbic acid in patients with venous ulcers.

**METHOD:**

longitudinal and descriptive quantitative study. During one year, 18 patients were assessed who were clinically diagnosed with venous ulcer in different stages, male and female, adult, with a mean injury time of 13 months. Ulmo honey was topically applied daily. The dressing was applied in accordance with the technical standard for advanced dressings, combined with the daily oral consumptions of 500 mg of ascorbic acid. The monitoring instrument is the assessment table of venous ulcers.

**RESULTS:**

full healing was achieved in 100% of the venous ulcers. No signs of complications were observed, such as allergies or infection.

**CONCLUSION:**

the proposed treatment showed excellent clinical results for the healing of venous ulcers. The honey demonstrated debriding and non-adherent properties, was easy to apply and remove and was well accepted by the users. The described results generated a research line on chronic wound treatment.

## Introduction

Vascular ulcers cause repercussions in different spheres. Besides affecting health, they worsen the quality of life of patients and their responsible caregivers. Their evolution is slow, their duration undefined and relapses can extend for months or years. These lesions are predominantly venous, with single lesions, presenting exudate, extensive, causing functional limitations in the victims and further multidimensional repercussions^(^ 1 ^)^. Ulcers cause a loss of workdays, early retirement and spending on prolonged treatment, with a great impact on the quality of life of the victims. The high prevalence of venous ulcer exerts a great impact in health resources. It is estimated that the management of venous ulcers uses between 1% and 3% of the annual budget in countries with developed health systems. In Chile, thousands of millions of pesos were spent in 2008^(^ 2 ^)^. 

In the 1960's, dressings started to be developed for chronic wound treatment in moist environments (APCAH), starting a rapidly changing career in the clinical research process, describing different treatments, growth factors, the application of microcurrent^(^ 3 ^)^, use of plasma rich in platelets for the healing of chronic ulcers in the lower limbs^(^ 4 ^)^. 

On the other hand, the interest in complementary therapies has increased in recent years, which has lead to the investigation of products traditionally known as beneficial for venous ulcer healing. Various papers have reported on the speed and effectiveness of wound healing treated with honey, besides its low cost. Studies have been developed to reintroduce its use in wound treatment, analyzing its anti-inflammatory, antibacterial and healing properties^(^ 5 ^-^ 7 ^)^. Multifloral bee honey has demonstrated its effectiveness to treat burns, pressure ulcers and wounds^(^ 8 ^-^ 9 ^)^. Recently, dressings have been developed for wound treatment based on Manuka honey from New Zealand (Bio18+Manuka Honey, Medihoney^(r)^ and L-Mesitran^(r))^. On the other hand, in Chile, the use of extract from monofloral Ulmo *(Eucryphia cordifolia*) honey has been patented with bactericidal and fungicidal properties^(^ 10 ^)^.

It is known that ascorbic acid is an essential factor in the regeneration process, as it promotes the proliferation of fibroblasts, AND synthesis and the mitochondrial metabolism, stimulates the development of the baseline membrane and reduces wound contraction^(^ 11 ^-^ 12 ^)^. The action of this vitamin has been studied in wound healing in guinea pigs through oral administration. The results showed that oral vitamin C improved the collagenization of the wound in comparison with a control group of animals (on a normal diet but without an extra dose of the vitamin studied)^(^ 13 ^)^. The action of oral vitamin C was studied in mice injuries, demonstrating that its administration increased the levels of tissue hydroxyproline, improving the tissue healing and the tensile strength at the scar level^(^ 14 ^)^.

Few studies have demonstrated the synergic effect of Ulmo honey associated with ascorbic acid. Our research team assessed this effect experimentally in type B burns in guinea pigs (*Cavia porcellus*), histologically observing its regenerative effects^(^ 15 ^)^. In this study, it was demonstrated that the unique use of Ulmo honey reduces the possibility of infection, inflammation and edema, leading to rapid healing. The formation of granular tissue, however, the activation of fibroblasts, formation of baseline membrane and keratinization were faster and better when the therapy was associated with ascorbic acid.

The role of the baccalaureate nurse should be highlighted, who is in charge of the dressings and should be trained for the management of wound care. This implies a standard criterion in the healing process and the comprehensive assessment of the injury evolution. To comply with these criteria, since the year 2000, the ministerial incorporation of a series of clinical guidelines has gained importance with a view to the management and treatment of wounds and ulcers^(^ 2 ^)^, in response to the need to train specialized monitors. According to article No. 113 of the Chilean Health Law^(16) ^and General Administrative Standard No. 19, "a baccalaureate nurse with technical background and competences should be in charge of the care management". In addition, specialization in institutions authorized to provide training is required, whether in foundations or universities.

In view of this problem, this study was developed, whose main objective was the clinical assessment of the effect of treatment using Ulmo honey and oral vitamin C in patients with venous ulcers.

## Method

### Study design and selection of patients

This study, which is part of the project DIUFRO DI13-0044, was developed at Hospital Intercultural de Makewe, Padre Las Casas, in the Región de La Araucanía, Chile. Between January and December 2012, 18 patients were assessed, all of whom were registered in the three-weekly outpatient healing program, male and female, with a mean age of 64 years, mean duration of the injury 13 months, clinically diagnosed as venous ulcer patients secondary to superficial venous failure without treatment by the hospital surgeon, who prescribed the treatment. The patients were hospitalized due to the rural area and the difficult access to their homes. They suffered from the following aggregated chronic illnesses: the two patients with type 1 venous ulcer (VU) suffered from arterial hypertension (AHT). Among the three patients with type 2 VU, one indicated diabetes mellitus II (DM-II) y dos AHT. For type 3 venous ulcers, one reported DM-II and 5 AHT. Among the cases of type 4 VU, one presented DM-II and two AHT. In total, three patients suffered from DM-II and 12 from AHT. 

To classify the venous ulcers, the assessment diagram established in the Clinical Guide for advanced comprehensive venous ulcer management^(^ 2 ^)^ was used, which specifies the classification of the following parameters: aspect of the ulcer, extension, depth, quantity of exudate, quality of exudate, necrotic tissue, granular tissue, edema, pain and surrounding skin. The possible results in increasing order of complexity are: Type 1 ulcer (10-15 points), Type 2 ulcer (16-21 points), Type 3 (22-27 points) and Type 4 (28-40 points). 

The Institutional Review Board of the Health Service Araucanía Sur, Chile (Protocol 1121) assessed the informed consent. After the patients had given their informed consent, the treatment started. For each patient enrolled in the dressing program, the medical history was collected and a clinical investigation was done, showing that the main location of these ulcers was the internal anterolateral and supramalleolar front of the leg. In patients with type 4 VU, the extension revealed a circular trend. Sixty-one percent of the patients with VU were hospitalized with a localized infection.

The melissopalynological analysis of the Ulmo honey selected for this study showed 91% of purity, determined as monofloral Ulmo honey according to Chilean standard NCh 2981.Of 2005. 

After selecting the patient, the protocol was applied in accordance with the technical wound dressing standards of the Chilean Ministry of Health. At the start of the treatment, the ulcer was digitally registered and the bacterial infection (if present) was appropriately controlled. For the dressings, Ulmo honey was topically applied and 500 mg of ascorbic acid was consumed orally each day. All patients showed an appropriate renal function. In addition, the treatment protocol included edema management using simple elastic compression and venous position, oral hydration using 2 liters of water per day and wetting of the skin surrounding the ulcer with neutral dermatological cream without alcohol. Six months after the healing of the wounds and termination of the treatment, the patient was received for outpatient control. The main criteria used to assess the wound were: presence of erythema and edema of the surrounding skin, presence of granular or necrotic tissue, applying the venous ulcer evaluation table every three weeks.

In the course of the treatment, each patient received "Self-care education on relapse prevention measures". The treatment sessions took place daily until the complete closure of the ulcers. The nurse specialized in comprehensive care for injured patients performed these actions.

To present and analyze the results, descriptive statistics were applied, including means and percentages, records in an Excel worksheet and display in a contingency table. 

## Results

The complete healing of the venous ulcers was achieved in all patients enrolled in the study. In the patients treated using the proposed protocol, no signs of complications were observed, such as allergies, spreading of the wound or infection. Patients hospitalized with a localized infection reported a mean subjective pain score of 5 on the Analogue Pain Assessment Scale (APA), without a direct relation between increased pain and the application of the honey. When the infectious process ceased, so did the pain.

After the monitoring period, five patients presented an ulcer relapse, after a mean period of six months, whose main cause was non-compliance with the post-discharge relapse prevention measures. The healing time according to the venous ulcer classification is indicated in Table 1. All lesions constituted epithelialization islets that soon gave way to healing bridges.

**Table 1 -:** Mean length of healing in days for each type of venous ulcer (VU) using topical treatment based on Ulmo honey and oral administration of ascorbic acid. Hospital Intercultural de Makewe, Padre Las Casas, Región de La Araucanía, Chile, 2012

VU classification	n	Mean (days)	SE
Type 1	2	20	0.89
Type 2	3	24	0.77
Type 3	8	45	1.1
Type 4	5	60	0.96

**Figure 1 -:**
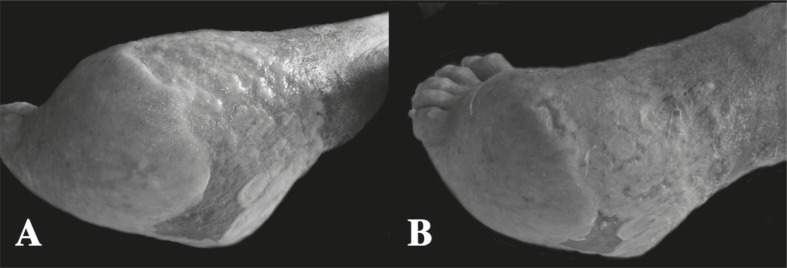
Treatment using Ulmo honey and ascorbic acid of type 3 venous ulcer in female patient. A. Initial wound with more than 75% of necrotic tissue, less than 25% of granular tissue and edema surrounding the ulcer (++). B. Wound after 20 days of treatment, less than 50% of necrotic tissue, 50% of granular tissue, edema surrounding the ulcer (+) and granulation and epithelialization bridges.

**Figure 2 -:**
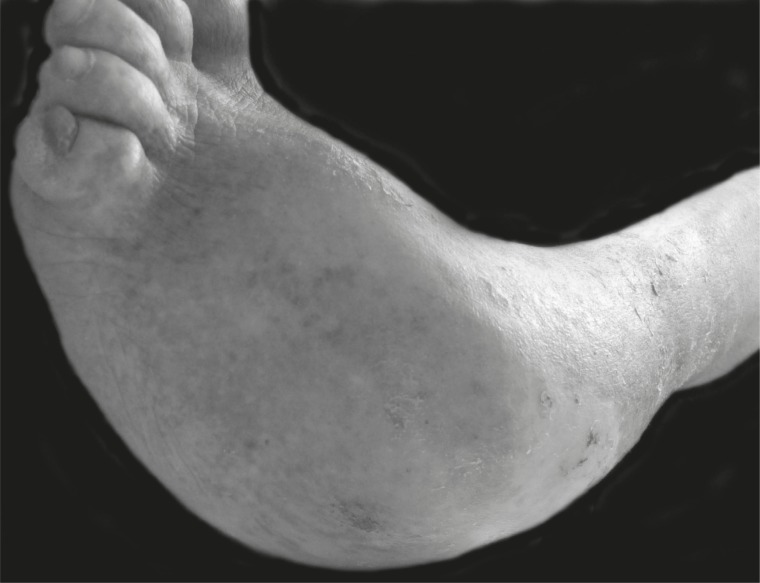
Final phase of the treatment using Ulmo honey and ascorbic acid of type 3 venous ulcer in female patient. The lesion was completely re-epithelialized, without formation of edema surrounding the ulcer.

**Figure 3 -:**
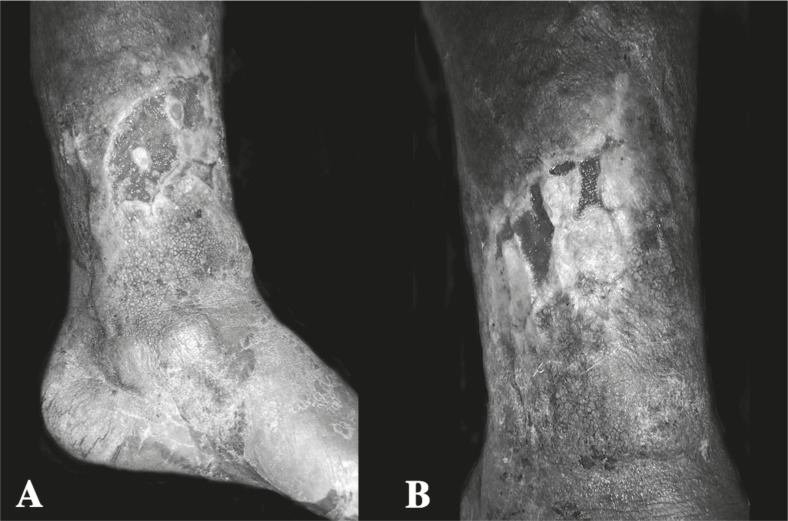
Treatment using Ulmo honey and ascorbic acid of type 3 venous ulcer in male patient. A. Initial wound showing infectious process with moderate exudate, eczematous and inflamed tissue surrounding the ulcer. B. Third week of treatment, ulcer with wide epithelialization bridges, granular tissue without infection, surrounding skin with reduced inflammation process.

**Figure 4 -:**
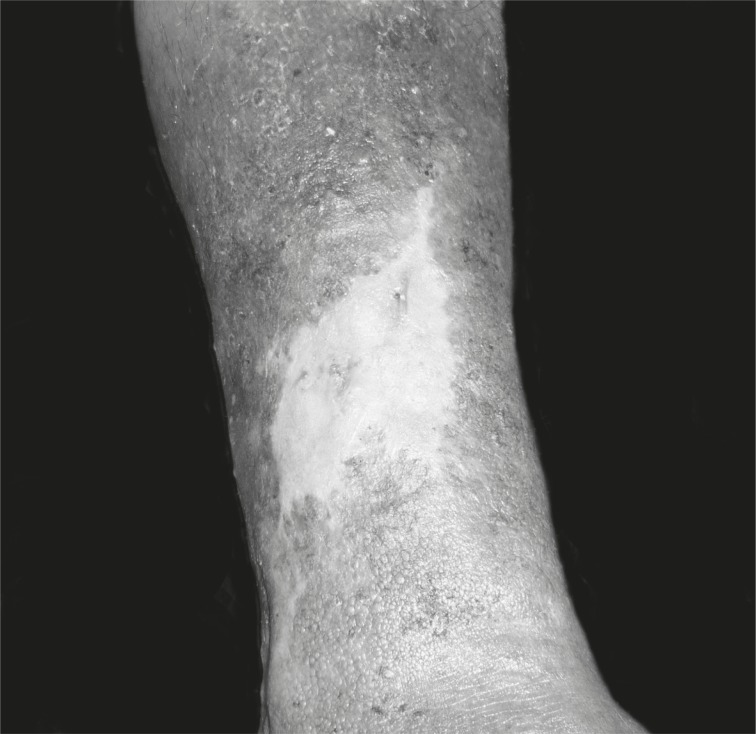
Final phase of treatment using Ulmo honey and ascorbic acid of type 3 venous ulcer in a male patient. Lesion 100% re-epithelialized

In Figures 1 and 2, images of the treatment based on Ulmo honey and ascorbic acid are shown for a female patient with type 3 venous ulcer (VU). The wound had existed for approximately one year. Twenty days after the treatment, the wound showed less than 50% of necrotic tissue. At the end of the treatment, the re-epithelialized area corresponded to 100%, without any edema surrounding the ulcer, and the patient was discharged after one month. In Figures 3 and 4, images of the treatment based on Ulmo honey and ascorbic acid are shown for a male patient with type 3 venous ulcer (VU). At the end of the treatment, the re-epithelialized area corresponded to 100% and the patient was discharged after one month. All treated patients showed a healing mechanism by secondary intention, with dressings applied according to the established protocol, without the need for dermoepidermal grafts. 

## Discussion

Venous ulcers are a common problem that represents a therapeutic challenge, as they provoke inability and consequently affect the quality of life. Similarly, they consume health system resources. This problem leads to the search for solution in therapies with cost-efficiency benefits. Our experience has demonstrated that treatment using Ulmo honey and oral vitamin C stimulates the formation of granular tissue and the proliferation of tissue adjacent to the ulcer, reducing the formation of tissue edema and reducing the local bacterial levels. Thus, rapid and good quality healing was achieved, besides effective care for the skin surrounding the lesion.

Venous ulcer treatments should achieve a good debridement of the wound to permit the formation of granular tissue and the consequent epithelialization. Several methods exist, including autolytic, chemical, mechanical and surgical debridement. In this study, the use of Ulmo honey showed excellent results in the autolytic debridement of venous ulcers. The debriding action of the honey could be due to the activation of proteases and metalloproteinases, which can be activated through oxidation processes, in combination with the inhibition of serines, thus supporting the wound debridement^(^ 17 ^)^.

No signs of infection appeared in the patients studies, which did happen in other treatments, such as autotransplantation, with a 3% infection rate^(^ 18 ^)^. To achieve this antibacterial action, the osmolarity, acidity and the presence of phytochemical compounds are important. In addition, the glucose oxidase enzyme present in honey is activated, catalyzing a slow production of H2O2, which inhibits the bacterial growth. This capacity varies among different types of honey. The main sugars in bee honey in general are fructose and glucose, with about 20% of humidity, proteins, vitamins and enzymes; it contains 35 minerals, lipids and flavonoids, besides another series of elements, among which germicide and inhibin stand out, which serve as natural antibiotics^(^ 19 ^)^. The honey from the Manuka tree (*Leptuspermun spp*.) is more effective when compared to treatment using Hydrogel. Its excellent antibacterial effects were demonstrated^(^ 20 ^)^, but comparative studies between Manuka and Ulmo (*Eucryphia cordifolia*) honey have showed that the latter achieved a better antibacterial effect for species like *Staphyloccus aureus*, *Escherichia coli* and *Pseudomonas aeroginosa* ^(^ 21 ^)^. The group of patients treated includes diabetic and hypertensive patients and patients with infected wounds. These were no reason for exclusion, nor did they affect the healing of the wounds treated, as these diseases were controlled and medically stabilized.

No evidence was found as to the best option for the topical treatment of venous ulcers. Most authors agree about the benefits of honey for wound healing, showing statistical evidence of its superiority in terms of the healing time when compared to other products^(^ 22 ^)^. Research suggests that the ideal alternative is a simple dressing, which is low-cost, non-adherent and acceptable to the patient^(^ 23 ^)^, properties of the proposed treatment based on Ulmo honey and vitamin C. The antioxidant capacity of bee honey has been endorsed. In addition, it activates the monocytic line, with the consequent release of cytokines, tumor necrosis factor alpha and interleukins^(^ 24 ^)^. If these qualities of honey are combined with the benefits of ascorbic acid, such as its great anti-inflammatory power, direct stimulation of collagen fiber synthesis and four-times higher proliferation of fibroblasts^(^ 25 ^)^, it could be inferred that we are boosting the honey-based treatment through the daily consumption of this vitamin.

## Conclusion

The proposed treatment achieves the expected clinical goal in accordance with the proposed objective and the literature review. This therapy evidenced excellent results, healing the wounds in 100% of the patients with all types of venous ulcers, with a mean 37 days of recovery, which is fast when compared to the time registered for conventional treatments. In combination, Ulmo honey and ascorbic acid can be considered ideal substances for the treatment of chronic wounds like venous ulcers. The honey shows antibacterial, debriding and non-adherent properties; it is easy to apply and remove and patients accept it because of its painless and smell neutralizing characteristics.

It is important to mention that, to achieve a faster and correct healing of venous ulcers, the application of a protocol-based dressing technique, the supervision of resting in the venous position, edema control through elastic compression and moisturizing of the skin surrounding the ulcer are favorable. In combination, these are part of nursing care management by the nurses specialized in wound care. Supported by an education plan based on the prevention of relapse, this permitted the successful treatment of these lesions. Finally, the clinical results described produced a histological research line in chronic wound treatment, using an alternative therapy of Ulmo honey combined with ascorbic acid.
